# Design and Application of Wuhan Ionospheric Oblique Backscattering Sounding System with the Addition of an Antenna Array (WIOBSS-AA)

**DOI:** 10.3390/s16060887

**Published:** 2016-06-15

**Authors:** Xiao Cui, Gang Chen, Jin Wang, Huan Song, Wanlin Gong

**Affiliations:** School of Electronic Information, Wuhan University, Wuhan 430079, China; cuixiao@whu.edu.cn (X.C.); wangjin1221@whu.edu.cn (J.W.); songhuan@whu.edu.cn (H.S.); gongwl@whu.edu.cn (W.G.)

**Keywords:** antenna arrays, HF radar, ionosphere, remote sensing

## Abstract

The Wuhan Ionospheric Oblique Backscattering Sounding System with the addition of an antenna array (WIOBSS-AA) is the newest member of the WIOBSS family. It is a multi-channel radio system using phased-array antenna technology. The transmitting part of this radio system applies an array composed of five log-periodic antennas to form five beams that span an area to the northwest of the radar site. The hardware and the antenna array of the first multi-channel ionosonde in the WIOBSS family are introduced in detail in this paper. An ionospheric detection experiment was carried out in Chongyang, Hubei province, China on 16 March 2015 to examine the performance of WIOBSS-AA. The radio system demonstrated its ability to obtain ionospheric electron density information over a wide area. The observations indicate that during the experiment, the monitored large-area ionospheric F2-layer was calm and electron density increased with decreasing latitude.

## 1. Introduction

Ionospheric research burgeoned in the 1920s when Appleton and Barnett of England confirmed the existence of the ionosphere. Since the end of World War II, a number of high-frequency (HF) radars and ionospheric research programs have been being pursued for measuring important characteristics and applications, such as long-range target monitoring, airborne early warning, and anti-stealth detection [1]. A number of HF radars and their achievements have been reported around the world, such as the new Digisonde-4D of the most famous digisonde family developed by the University of Massachusetts Lowell Center for Atmosphere Research (ULCAR) [2], the Advanced Ionospheric Sounder developed at the National Institute of Geophysics and Volcanology (AIS-INGV) in Italy [3], the DAMSON-a low power Doppler and multipath sounding network in the UK [4], the Canadian Advanced Digital Ionosonde (CADI) digital ionosondes [5], the Jindalee over-the-horizon radar (OTHR) of Australia [6], the TIGER radar of the Super Dual Auroral Radar Network (SuperDARN) network [7], the French OTH radar NOSTRADAMUS [8], *etc*.

Cooperation between the Electronic Information School Wuhan University (Wuhan, China) and University of Paris-Sud (Paris, France) in the fields of ionosphere physics and transmission characteristics of high-frequency channels across Eurasia has been carried out since 1985. In reference to the experience from the STUIO5 system [9], Wuhan University began to develop ground-based ionospheric sounding systems for ionospheric research and HF radio propagation in 2001. The prototype of WIOBSS (Wuhan Ionospheric Oblique Backscattering Sounding System) was developed successfully in 2003. The WIOBSS received the first vertical incident echoes in May 2004 and recorded the first sweep frequency ionogram for backscatter in September 2004 [10]. Initially, the WIOBSS was a monostatic digital ionosonde based on the VXI-Bus (VME eXtensions for Instrumentation). A remote digital receiver with Global Positioning System (GPS) for frequency and clock synchronization was developed to work together with the WIOBSS in 2008 [11]. The receivers can be used for ionospheric bistatic backscatter sounding, oblique incident sounding, as well as sky-wave sea-state sounding. Many kinds of waveforms have been tested on the WIOBSS and the ionograms obtained by different waveforms have been compared and reported [12]. In 2010, the Universal Serial Bus (USB) and high-performance field programmable gate arrays (FPGA) were applied in the new system. The previous radio systems in the WIOBSS family were all single-channel systems, which were applied to observe the ionospheric E-layer, F-layer, and E-region field-aligned irregularities [13,14]. They have the ability to measure the echo amplitude, range, and Doppler shift [15,16], and are used to investigate electron density variations and travelling ionospheric disturbances [17,18].

To monitor the large-area ionosphere, a new generation of the WIOBSS that incorporate a log periodic antenna array (WIOBSS-AA) is presented in this paper. Firstly, we introduce the hardware frames of the WIOBSS-AA, including the data/control routing solutions, the radio frequency (RF) analog front end, the digital transceiver and the log-periodic antenna array, and so on. Then we outline the new features of the data processing and the digital beam-forming and, finally, make a conclusion based on the initial results recorded by WIOBSS-AA.

## 2. System Description

**Figure 1:**
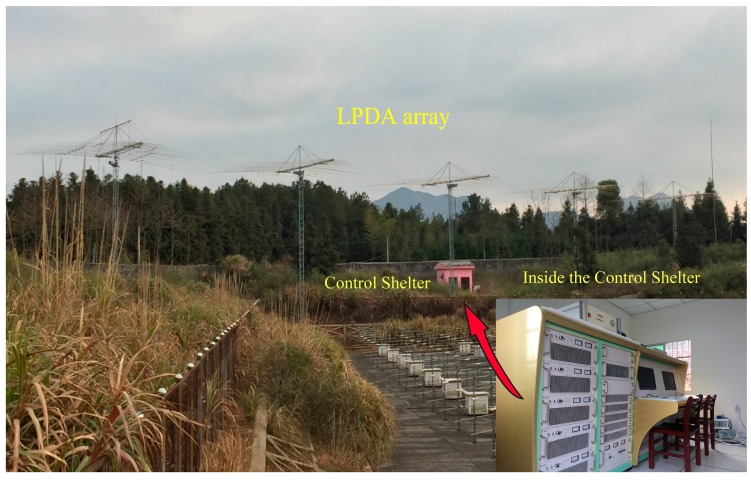
Photo of WIOBSS-AA site showing the log-periodic antenna array. The pink building in the center of the photo is the shelter of the hardware. The mainframe of the WIOBSS-AA is displayed in the lower right corner.

The development of the WIOBSS has benefited from the ascent of digital techniques since the 2000s. There have been a lot of changes and upgrades on the hardware and system architecture of the WIOBSS in recent years. The WIOBSS-AA is the latest system of the WIOBSS family, which is also a totally functionally-upgraded system. The most significant change is the application of the log periodic antenna array, which is located in Chongyang, Hubei province, China (29.53° N, 114.14° E), as shown in Figure 1. The antenna array was established in May 2013. The hardware and software of the radar system was accomplished in August 2014 and placed in the pink house in the center of Figure 1. The mainframe of WIOBSS-AA is displayed in the lower right corner of this figure. 

**Figure 2:**
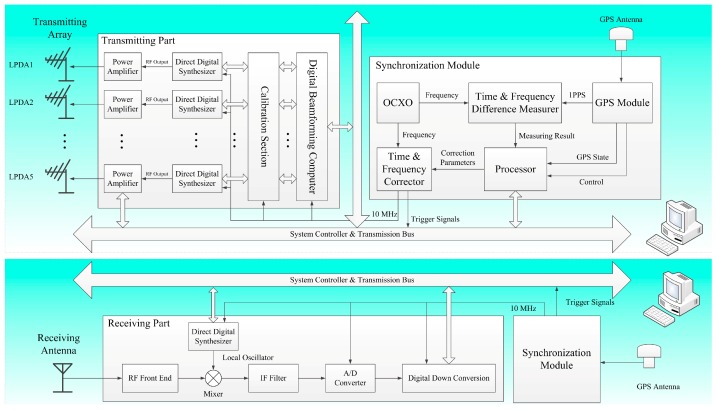
General architecture of WIOBSS-AA. This radio system is composed by the transmitting part, receiving part, and synchronization module.

The radio system block diagram consists of three parts: the transmitting part, the receiving part and the synchronization module, as shown in Figure 2. The transmitting part is composed of five channels shown in the above schematic and the receiving part is a single channel system in the schematic below. The transmitting and receiving parts share a time-frequency synchronization module, which is based on the GPS system and used for the time and reference frequency synchronization between the operating parts. The USB bus is applied in the transmitting and receiving parts, as well as the synchronization module. The core of the USB is the CY7C68013 unit, which belongs to the family of USB 2.0 transceivers. 

### 2.1. Transmitting Part

The function of the transmitting part is generating all kinds of modulated RF waveforms according to a set program. The multi-channel technology and digital beam-forming are applied in the transmitting part. The five-channel transmitting part is based on the AD9911 chip. This chip is a complete direct digital synthesizer with low power dissipation and high performance. The digital data moves to the digital beam-forming computer through the USB bus. The phase and amplitude of the digital waves are adjusted precisely in the digital beam-forming computer and synthesized into five channels for beam formation. A calibration section is designed to calibrate the amplitude-frequency and phase-frequency response in the digital domain for consistency across the five channels. The digital beam-forming computer and the calibration section will be explained in detail in the following section. The generated waveforms are amplified to 50 dBm and then emitted by each log-periodic antenna.

### 2.2. Receiving Part

The receiving part is a single-channel system, including the RF front end, the mixer, the A/D converter, and the digital down conversion (DDC). The RF front end is designed with superheterodyne architecture and consists of an RF switch, suboctave filters, and amplifier. The suboctave filter group is used to avoid out-of-band noise and multiple-frequency signal interference. In the receiving channel, a single intermediate frequency (IF) of 41.4 MHz and band-pass sampling techniques are applied to simplify the hardware structure. The DDC module consists of a Hilbert transformer, a cascaded integrator comb filter (CIC), and a finite impulse response (FIR) digital filter, which is embedded in a FPGA [19].

### 2.3. Synchronization Module

The WIOBSS-AA loads a time-frequency synchronization module with a GPS core. The GPS is used to calibrate the system clock. The synchronization unit delivers a long-term stability of 1 pulse per second (1PPS) and a 10 MHz clock generated by a local oven-controlled crystal oscillator (OCXO) to the FPGA. The precision-unknown 10 MHz clock is calibrated by the 1PPS scale in the FPGA. The calibrated clock is used for the reference clock in order to maintain frequency synchronization in the entire system. The transmitting and the receiving parts both use the synchronization module for clock and reference frequency synchronization.

## 3. Digital Beam-Forming

Digital beam-forming (DBF) is a marriage between antenna technology and the evolution of digital devices. It is an advanced approach to steer phased array antennas. The synthetic narrow beam enhances the directional resolution of the radio system. The number of array elements and space between them determine the beamwidth and level of sidelobes [20]. The directional pattern of a uniform line array can be expressed as:

(1) $$\left|F(\theta )\right|=N\frac{\sin(\frac{N\pi }{\lambda }d(\sin\theta -\sin\theta_{B}))}{\frac{N\pi }{\lambda }d(\sin\theta -\sin\theta_{B})}$$

where *F*(θ) is the array pattern, θ is the azimuthal angle, *d* stands for the distance between adjacent antennas, λ is the wavelength of the transmitting signal. *N* is the number of array elements, θ*_B_* is the maximum value of the array beam pointing in azimuthal direction. The array pattern is represented as the sinc function, and the maximum value is *N* [21].

Phased array antennas are used to steer a narrow beam over an arc from a fixed antenna array. When |*F*(θ)| = 1 , the maximum value of the array beam pointing is at θ*_B_* (θ *=* θ*_B_*):

(2) $$\theta_{B}=\arcsin(\frac{\lambda }{2\pi d}\Delta \phi_{B})$$

where Δ$\phi_{B}$ is the phase difference between adjacent antennas and can be changed to steer the array main beam direction. At each transmitter the direction is adjusted with the application of a systematic phase delay to the signal sent to the antenna.

### 3.1. Log-Periodic Dipole Antenna

**Figure 3:**
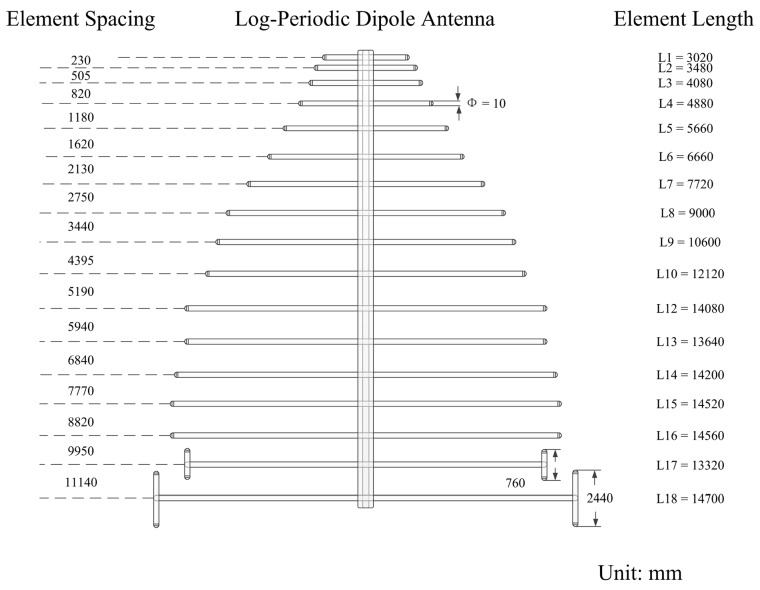
Sketch of the LPDA of the WIOBSS-AA.

The antenna in the array is the Log-Periodic Dipole Antenna (LPDA). The structure of the antenna is illustrated in Figure 3. The LPDA unit consists of 18 dipole elements with a boom length of approximately 14 m, while the longest dipole element is almost 15 m long. All of the elements are made with 10-mm-diameter aluminum tubes. The LPDA is top fed and the feed point is near the shortest dipole element. The 18 dipole elements are fed via a transmission line. The antenna balun is made of coiled coaxial cable, which is installed on the main rod. 

**Figure 4:**
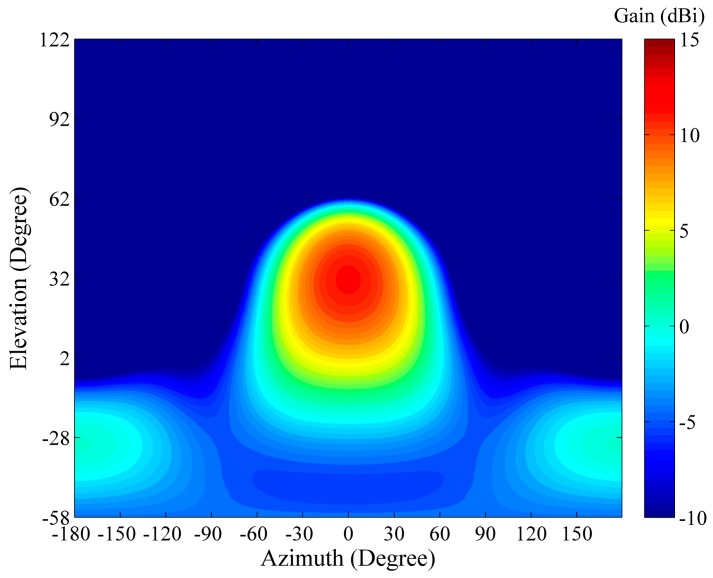
Computed radiation pattern of a single LPDA at 10 MHz (main lobe vector alignment), where dBi values are with reference to an isotropic gain pattern.

The antenna of the WIOBSS-AA was designed and validated by the Computer Simulation Technology MICROWAVE STUDIO (CST MWS) (Bad Nauheimer Strasse, Darmstadt, Germany). Each element of the LPDA is formed by wires in the model. The LPDA is excited by a voltage source. The simulations are performed for ground environment using lossless ground approximation. The computed radiation pattern of a single LPDA is shown in Figure 4. 

The symmetric antenna radiation pattern of a single LPDA pointing to elevation of 32° has a half power beam width of 71.4°. The directive gain of the LPDA is 11.7 dBi.

### 3.2. Log-Periodic Dipole Antenna Array

The transmitting antenna array of the WIOBSS-AA consists of five log-periodic dipole antennas. These antennas are arranged in a line and the distance between adjacent antennas is 15.3 m. The height of each antenna is 12 m. It is well known that, in phased linear arrays, the optimum antenna separation for beam forming is λ*/2* [22]. For the WIOBSS-AA frequency range of 6–30 MHz, this results in an optimum spacing between 5 m (at 30 MHz) and 25 m (at 6 MHz). Due to the length of the longest dipole element and the limited space, the antenna separation is selected as 15.3 m. There are five transmitting channels and the channels are independent of each other. Each transmitting channel is mainly comprised of the DDS device and a 2 kW solid state power amplifier. The output of each power amplifier is connected to the antenna. Based on the high-speed direct digital frequency synthesizer, the phase, as well as the amplitude difference between each transmitting channel, can be adjusted by modifying the phase offset register and amplitude register. The accuracy of the phase is about 0.02 degree (the phase offset resolution of AD9911 is 14-bit) and the accuracy of the amplitude is about 0.5 mV (the amplitude-scaling resolution of AD9911 is 10-bit).

**Figure 5:**
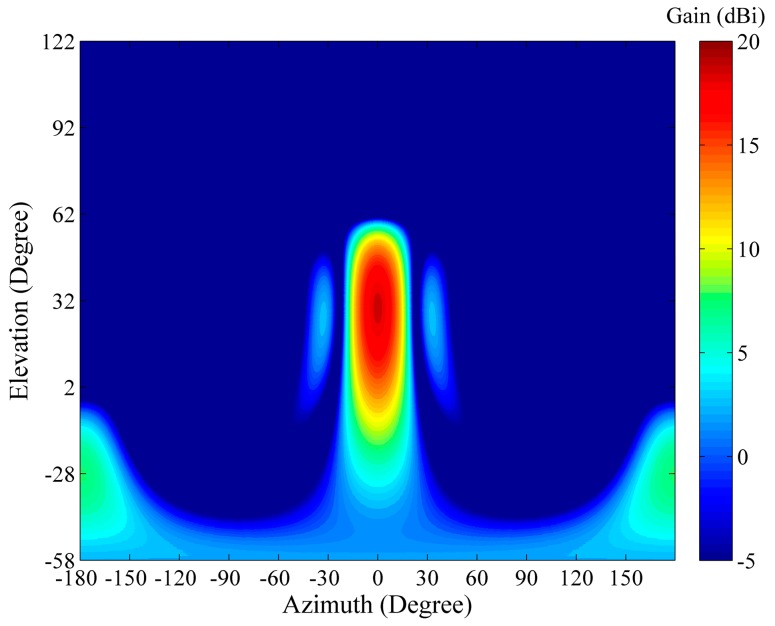
Computed radiation pattern of the antenna array at 10 MHz (main lobe vector alignment), where dBi values are with reference to an isotropic gain pattern.

**Figure 6:**
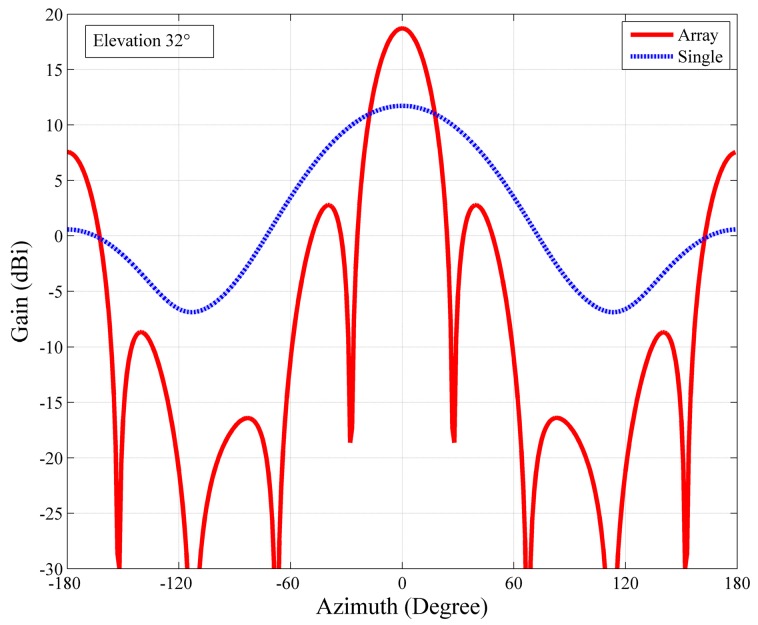
Comparison of the radiation patterns between a single antenna (blue line) and the antenna array (red line) at 10 MHz (main lobe vector alignment), where dBi values are with reference to an isotropic gain pattern.

The computed radiation pattern of the whole array is shown in Figure 5. The symmetric antenna radiation pattern of the full array pointing to an elevation of 32° has a half power beam width of 22.8°, a directive gain of 18.7 dBi, and an almost symmetric first side-lobe with more than 15 dB suppression with respect to the main lobe. Although the log periodic antenna is a directional antenna, it has a large beam width of 71.4°, as shown in Figure 6 (blue dashed line). Compared with a single antenna, the antenna array has a relatively narrow beam width of only 22.8° and higher gain, as shown in Figure 6 (red solid line).

**Figure 7:**
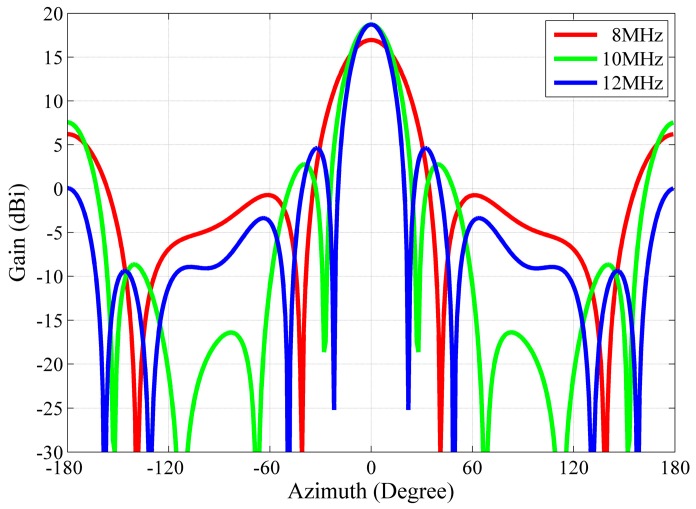
Comparison of the antenna array radiation patterns at 8 MHz (red line), 10 MHz (green line), 12 MHz (blue line) (main lobe vector alignment), where dBi values are with reference to an isotropic gain pattern.

**Figure 8:**
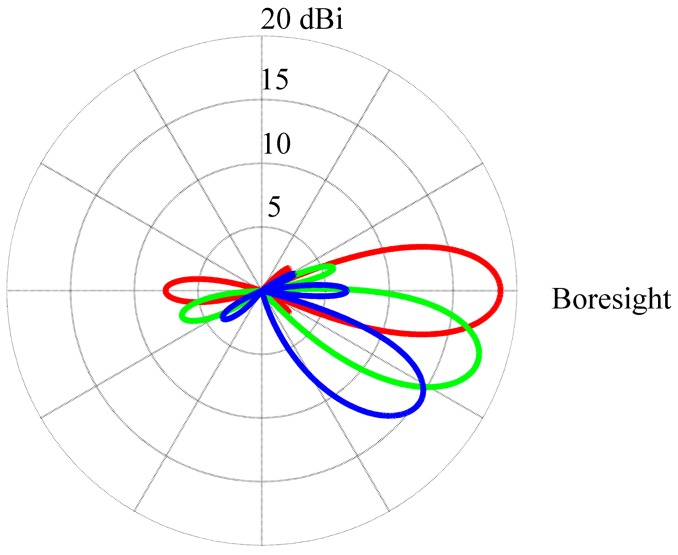
Azimuthal total radiating field plots for the five-beam antenna array phased at 0° (red line), 18° (green line), and 36° (blue line) from the boresight. The operating frequency is 10 MHz and the elevation angle is 32°. The boresight is to the right of the Figure. The circular grid labels are with reference to the outer ring value, where dBi values are with reference to an isotropic gain pattern.

How changing the frequency affects the beam pattern of the array is demonstrated in Figure 7. The antenna radiation pattern of the antenna array has a half power beam width of 32.3° at 8 MHz (red line), 22.8° at 10 MHz (green line), and 19° at 12 MHz (blue line). The width of the beam decreases with the increased frequency. Unfortunately, the sidelobe and backlobe rise with decreased frequency. The beam pointing of the array has different elevation for different frequency. The elevation angle is 42° at 8 MHz, 32° at 10 MHz, and 30° at 12 MHz. With an increase in frequency, the elevation of the beam decreases. The 15.3 m antenna separation decides the optimum operating frequency is 10 MHz. The antenna separation limits the beam steerability [23]. Large grating lobes start appearing when the beam is steered beyond ±36° from the boresight, as shown in Figure 8.

## 4. Array Calibration

The realization of a digital beamforming phased array has several technological challenges. The most important one is phase synchronization and amplitude equality across the RF transmitting channels. For engineering calibration methods, at present, there are two major types of array calibration algorithms: passive calibration algorithm [24] and active calibration algorithm [25,26]. In WIOBSS-AA, we focus on the reverse application of the term multiple signal classification (MUSIC) [27] and develop a new engineering calibration algorithm based on eigenvalue decomposition.

Assume that a single signal is picked up by a multiplex parallel array in one incident direction. There is the following relation between the amplitude, phase error vector of each channel *Γ* and received data *X*(*t*):

(3) X(t)=Γa(θ)s(t)+n(t)

where *a*(θ) stands for the array, *s*(*t*) stands for the signal space, and *n*(*t*) stands for the noise space. The principle of MUSIC is to estimate the signal space *s*(*t*) using the known *Γ*, *a*(θ)and *X*(*t*). However, when we know the signal space *s*(*t*), the joint estimation to *Γ* could be carried out using *s*(*t*), *a*(θ), and *X*(*t*). The covariance matrix *R* of *X*(*t*)can be expressed as:

(4) $$R=\sigma_{s}^{2}\Gamma a(\theta_{s})a^{H}(\theta_{s})\Gamma +\sigma^{2}I$$

where *a^H^* is the conjugate transpose matrix of *a*, θ*_s_* is a specific incident azimuth (here θ*_s_* = 0), σ*_s_^2^* is the incident signal power, σ*^2^* is the noise power, and *I* is the identity matrix. The signal subspace vector *e* can be obtained by making eigenvalue decomposition of *R*:

(5) Γa(0)=ke

where *e* is the eigenvectors corresponding to the largest eigenvalues of *R* matrix, and *k* is a pending complex constant.

(6) $$a(0)={\left[1,1,...,1\right]}^{T}$$

(7) Γ=ke

Channel one is selected as a reference channel:

(8) $$\left[\begin{array}{c} 1\\ \Gamma_{2}\\ \begin{array}{l} .\\ .\\ . \end{array}\\ \Gamma_{m} \end{array}\right]=\left[\begin{array}{c} ke_{1}\\ ke_{2}\\ \begin{array}{l} .\\ .\\ . \end{array}\\ ke_{m} \end{array}\right]$$

So:

(9) $$k=\frac{1}{e_{1}}$$

(10) $$\Gamma_{i}=ke_{i}(i=2,3,...,m)$$

*Γ_i_* is a complex constant that can be expressed as *Ae*${}^{j\varphi }$, *A* indicates the amplitude difference among channels and φ indicates the phase difference among channels. 

**Figure 9:**
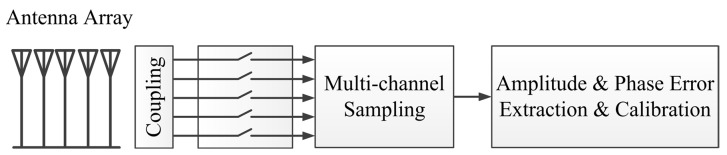
Calibration section of the WIOBSS-AA.

The intrinsic amplitude and phase difference among channels caused by antennas, feed cables, as well as transmitting channels could be figured out through this method. In the WIOBSS-AA, the couplers are installed at the root of each antenna, as shown in Figure 9. All of the transmitting channels are calibrated by setting the amplitude registers and the phase offset registers of AD9911 to obtain great amplitude and phase consistency before monitoring. The whole array calibration consists of two steps, the first step is internal calibration with small calibration signals that are routed to the couplers and then through multichannel sampling, so that the amplitude and phase error can be extracted and calibrated. The second step is the far-field calibration, which is a similar step using a source in a known location. The error of the antenna system is calibrated by the second step. In the experiment, the inherent error of the antenna is relatively fixed. Thus, there is no need to repeat step two in many cases.

## 5. Ultra-Wide Ionospheric Monitoring

Oblique backscatter sounding is a powerful tool for large-area ionospheric detection. The WIOBSS-AA applies the backscatter technology to monitor the northwest ionosphere. The oblique backscatter swept-frequency ionograms recorded by the WIOBSS-AA provides the amplitudes of backscatter echoes with respect to group distance against operating frequency. Applications of the inversion algorithms to the backscatter ionograms can extract the useful information regarding the ionospheric electron density distribution along the propagation paths. Some oblique backscatter sounding results of WIOBSS-AA are shown in this section. 

### 5.1. Experiment Facilities and Results

On 16 March 2015, a large-area ionospheric monitoring experiment was carried out in Chongyang. During the experiment, WIOBSS-AA periodically transmitted the phase-coded pulse train waveform and received the echoes during the pulse interval. The applied waveform parameters of the radio system were 39.0625 kHz pulse repetition rate, 20% duty cycle, and 511-bit pseudo-random code used as the modulating signal [28,29]. The range resolution was 3.84 km. The unambiguous Doppler measuring range was between [−3.81 Hz, +3.81 Hz] and the Doppler resolution was 0.0596 Hz. WIOBSS-AA was operated in the swept-frequency mode. The initial, end, and step frequencies were 6.6 MHz, 13.2 MHz, and 100 kHz, respectively. 

**Figure 10:**
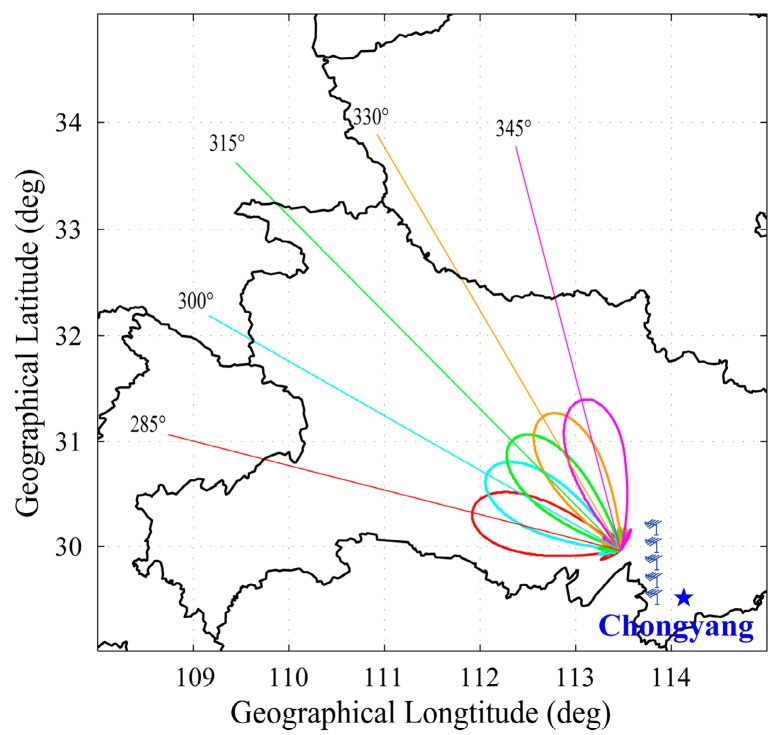
Map showing the location of the WIOBSS-AA in Chongyang, Hubei province, China and the antenna pointing in the experiment of 16 March 2015. The five main beams of the WIOBSS-AA are denoted by the different colors.

The location, as well as the five beam directions, of WIOBSS-AA is shown in Figure 10. The log-periodic antennas pointed northwest. Due to being a phased antenna array, the WIOBSS-AA is able to cover the large-area ionosphere with only 500 W peak power. The five beams pointed to 285°, 300°, 315°, 330°, and 345° in a clockwise manner. The beam elevation of the antenna array decreases with increasing operating frequency. The elevation is 42°, 32°, and 30° at 8, 10, and 12 MHz operating frequency, respectively. A receiver nearby with a broadband folded dipole antenna was used to record the echoes of the transmitting array. 

**Figure 11:**
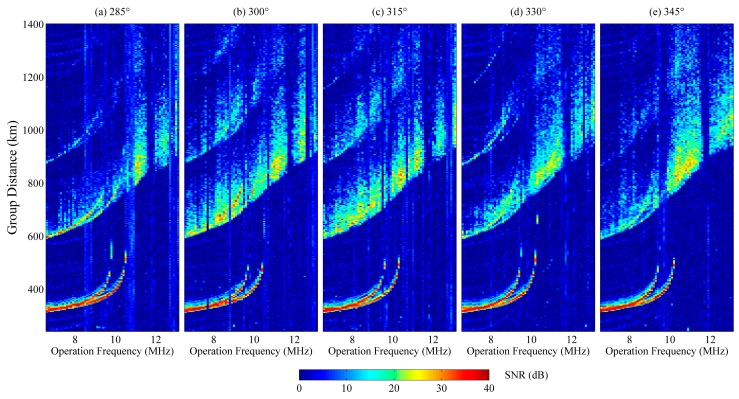
Swept-frequency oblique backscatter ionograms of WIOBSS-AA with five different beams, (**a**) 285°, (**b**) 300°, (**c**) 315°, (**d**) 330°, and (**e**) 345° in a clockwise manner. The operating frequency is from 6.6 to 13.2 MHz with 100 kHz step. The detecting range is from 100 to 1400 km with 3.84 km range resolution. The first swept-frequency ionogram began to be recorded at 9:40 L.T. and the last ionogram was obtained at 9:56 L.T. The color bar represents the signal to noise ratio (SNR) of the recorded echoes.

**Figure 12:**
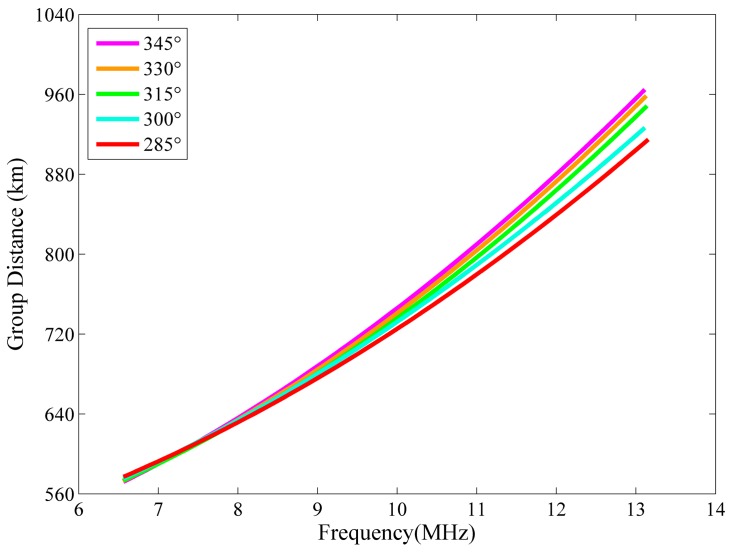
The leading edges of the oblique backscatter swept-frequency echoes of the five beams.

The ionograms of the five beams shown in Figure 11 all depict the distribution of scattered power as a function of group distance. Strong radio-frequency interference in the ionograms was removed [30]. The signals between 200 and 580 km are the vertical reflected echoes from F2-layer. The oblique backscatter echoes appear from 600 to 1400 km. The two-hop vertical-incidence echoes were overlapped with the oblique-incidence backscattered echoes. Usually, the range of the two-hop vertical-incidence echo is double that of the one-hop echo. So, by assessing the one-hop echo trace, the two-hop echo trace can be removed and then the leading edge of the backscattered echoes can be well recognized. Due to focusing effects, the backscatter echoes have an obvious smooth leading edge at the minimum distance of each operating frequency. The leading edge of each beam is extracted and displayed in Figure 12. Initially, the leading edges are overlapped. With an increase of the operating frequency, the detecting area of each beam is further and further from each other, and the leading edges also gradually separate from each other. The further north the beam points, the higher the leading edge is in the group range. 

### 5.2. Inversion of the Backscatter Ionograms

The inversion of the backscatter ionograms is based on the QP model [31,32]. The initial parameters of QP model are determined by the International Reference Ionosphere (IRI) model [33], as well as the real-time recorded electron density profiles of Wuhan digisonde. The leading edges of backscatter ionograms are segmentally fitted to obtain the ionospheric characteristics on each radio path as realistically as possible. The inversion method makes full use of the recorded backscatter ionograms, which are based on the simulated annealing algorithm [34]. By dividing the leading edge into several groups, this method can extract ionospheric information of different areas along the sounding path and eventually provide useful details of ionospheric parameters [35]. 

**Figure 13:**
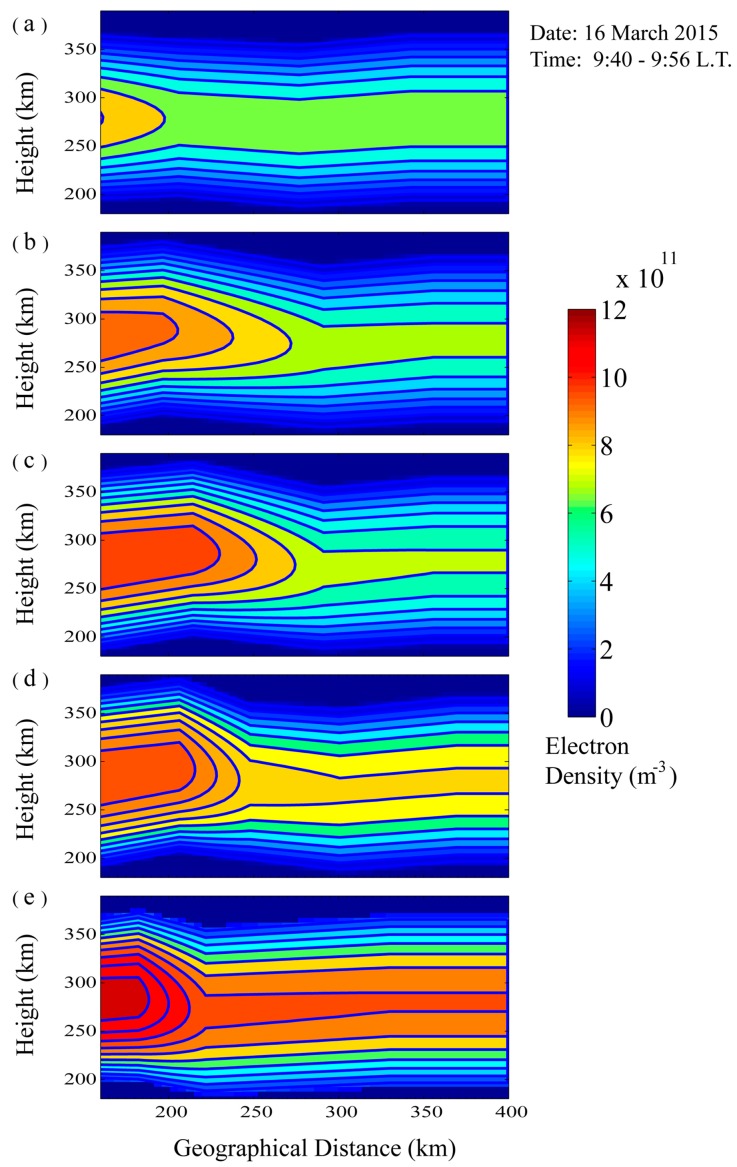
The two-dimensional ionospheric electron density distributing plots of five different directions corresponding to the five beams of the antenna array. The electron density distributing plots (**a**–**e**) is inverted by the leading edge of the beam of 345°, 330°, 315°, 300°, and 285°, respectively.

**Figure 14:**
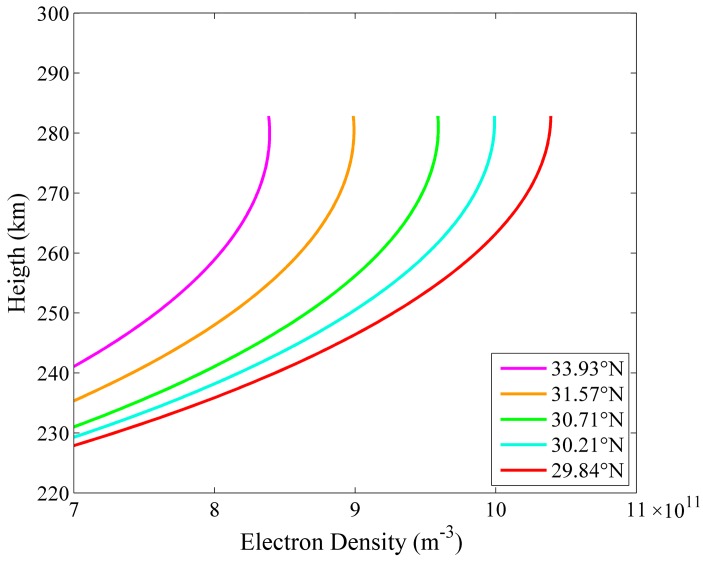
The electron density profiles in the five locations of the same longitude (112.77° E) and different latitudes (29.84° N, 30.21° N, 30.71° N, 31.57° N, 33.93° N).

As shown in Figure 13, the inverted electron density profiles distributed along the detecting distance of the five beams are very different. The five electron density profiles of the same longitude and different latitude are selected from Figure 13 and displayed in Figure 14. We find that in the experimental period the electron density at the 112.77° E longitude increased with decreasing latitude. 

**Figure 15:**
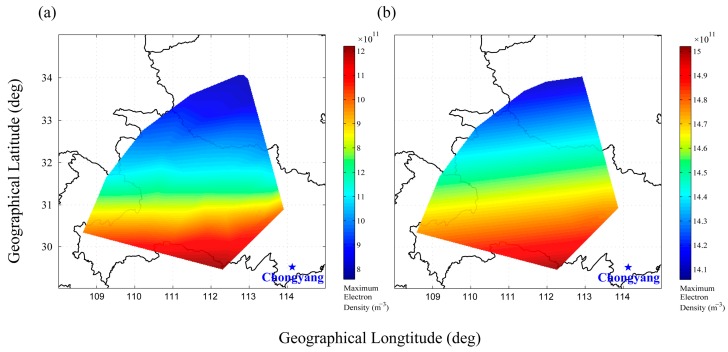
Comparison of the maximum electron density maps between the inversion results obtained from the five two-dimensional ionospheric electron density distributing plots (**a**) and the IRI-2012 model (**b**).

The maximum electron density in the two-dimensional ionospheric electron density plots of the five beams is extracted and then used to compose the maximum electron density map over a fan-shaped region as shown in Figure 15a by applying a two-dimensional interpolation algorithm. Over the observing region, lower maximum ionospheric electron density is found in the more northerly region. A longitude difference in the electron density distribution is not obvious. Using the IRI-2012 model data, the similar result is also gotten, as shown in Figure 15b. The variation range of ionospheric electron density in the F region is smaller in the IRI-2012 model than that in the inversion results. The IRI model is based on experimental evidence using all available ground and space data sources. Although there are no ionospheric stations in the observing area of WIOBSS-AA, IRI model is useful for the evolving theoretical understanding of ionosphere. The F2 layer equatorial anomaly, the “fountain effect”, is strong in the Asia region and the “northern crest” is at 20°–30° N. That is the most possible reason why the electron density drops for higher latitude values in Figure 15.

## 6. Conclusions

This present paper describes the design and application of the WIOBSS-AA. It is the first multi-channel radio system applying the log-periodic antenna array in the WIOBSS family. It can monitor the large-area ionosphere with five antenna beams and provide the electron density information over the observed region. The electron density profiles and the two-dimensional maximum electron density map over the observing region can be obtained only with the WIOBSS-AA radio system. More log-periodic antennas will be added to increase the azimuth resolution. The measuring technique for the echo elevation angle is also in development. It will be very important for improving the inversion of the ionospheric electron density.
